# Dynamics of Cognitive Impairment in MCI Patients over a Three-Year Period: The Informative Role of Blood Biomarkers, Neuroimaging, and Genetic Factors

**DOI:** 10.3390/diagnostics14171883

**Published:** 2024-08-28

**Authors:** Irina Morozova, Yana Zorkina, Alexander Berdalin, Anna Ikonnikova, Marina Emelyanova, Elena Fedoseeva, Olga Antonova, Dmitry Gryadunov, Alisa Andryushchenko, Valeriya Ushakova, Olga Abramova, Angelina Zeltser, Marat Kurmishev, Victor Savilov, Natalia Osipova, Irina Preobrazhenskaya, Georgy Kostyuk, Anna Morozova

**Affiliations:** 1 Mental-Health Clinic No. 1 Named after N.A. Alekseev, 115191 Moscow, Russia; zorkina.ya@serbsky.ru (Y.Z.); alex_berdalin@mail.ru (A.B.); alissia.va@mail.ru (A.A.); ushakovavm@yandex.ru (V.U.); abramova1128@gmail.com (O.A.); angelinazeltser@yandex.ru (A.Z.); 5086773@mail.ru (M.K.); vsavilov@mail.ru (V.S.); nata.osipoff@yandex.ru (N.O.); kgp@yandex.ru (G.K.); hakurate77@gmail.com (A.M.); 2 V. Serbsky National Medical Research Centre of Psychiatry and Narcology, 119034 Moscow, Russia; 3 Center for Precision Genome Editing and Genetic Technologies for Biomedicine, Engelhardt Institute of Molecular Biology, Russian Academy of Sciences, 119991 Moscow, Russia; anyuik@gmail.com (A.I.); emel.marina85@gmail.com (M.E.); elfed0@mail.ru (E.F.); markelka@rambler.ru (O.A.); grad@biochip.ru (D.G.); 4 Department of Mental Health, Faculty of Psychology, M. V. Lomonosov Moscow State University, 119991 Moscow, Russia; 5 Department of Nervous Diseases and Neurosurgery, I. M. Sechenov First Moscow State Medical University (Sechenov University), 119435 Moscow, Russia; 6 Department of Psychiatry and Psychosomatics, I. M. Sechenov First Moscow State Medical University (Sechenov University), 119435 Moscow, Russia; 7 Department of Psychiatry, Federal State Budgetary Educational Institution of Higher Education Russian Biotechnological University, 125080 Moscow, Russia

**Keywords:** MCI, polygenic risk, genetic risk, APOE, MMSE, MOCA, cognitive decline, long-term study

## Abstract

Given the high growth rates of cognitive decline among the elderly population and the lack of effective etiological treatments, early diagnosis of cognitive impairment progression is an imperative task for modern science and medicine. It is of particular interest to identify predictors of an unfavorable subsequent course of cognitive disorders, specifically, rapid progression. Our study assessed the informative role of various risk factors on the dynamics of cognitive impairment among mild cognitive impairment (MCI) patients. The study included patients with MCI (*N* = 338) who underwent neuropsychological assessment, magnetic resonance imaging (MRI) examination, blood sampling for general and biochemical analysis, *APOE* genotyping, and polygenic risk score (PRS) evaluation. The *APOE ε4/ε4* genotype was found to be associated with a diminished overall cognitive scores initial assessment and negative cognitive dynamics. No associations were found between cognitive changes and the PRS. The progression of cognitive impairment was associated with the width of the third ventricle and hematological parameters, specifically, hematocrit and erythrocyte levels. The absence of significant associations between the dynamics of cognitive decline and PRS over three years can be attributed to the provided suitable medical care for the prevention of cognitive impairment. Adding other risk factors and their inclusion in panels assessing the risk of progression of cognitive impairment should be considered.

## 1. Introduction

One of the most important tasks facing modern science and health is the study and prevention of cognitive impairment in the aged population. The World Health Organization estimates that the number of people aged 60 and over will increase from 1 billion in 2020 to 1.4 billion in 2030 [1]. Senile asthenia, which accompanies natural aging and affects sensitivity to stressors and immune system function, significantly impacts cognitive resilience [2,3].

Over the past 30 years, researchers have increasingly focused on the condition that lies between healthy aging and dementia, referring to it as mild cognitive impairment (MCI) or mild neurocognitive disorder. In 2003, the diagnostic criteria for MCI were first established, remaining relevant today [4,5].

According to the current data, the incidence rate of MCI among individuals aged 60 and over exceeds 15% [6,7]. However, not every case of MCI progresses to clinical dementia. Not uncommonly, patients diagnosed with MCI are reclassified, demonstrating a state of reduced cognitive function during normal aging. Despite this, long-term follow-up research shows that 50–60% of such cases regress to MCI and have a 5–6 times higher risk of dementia [8].

Since MCI is characterized by a high risk of transitioning directly to dementia, in particular, to Alzheimer’s disease (AD) [9], the importance of timely diagnosis and preventive measures against this pathology is undeniable. Moreover, the timely prevention of cognitive impairments can significantly delay the onset of pathological symptoms [10].

One of the potential ways to improve the early diagnosis of dementia is the development of biomarker-based preventive interventions. The validation of biomarkers associated with cognitive impairments remains relevant. Factors affecting the dynamics of cognitive decline are divided into modifiable and non-modifiable.

In addition to the significant influence of genetic factors, it is important to consider modifiable risk factors that are associated with cognitive decline. Neuropathological processes occurring in MCI can lead to a decrease in the volume of brain structures [11,12,13], disruptions in functional connectivity [14,15], and ventricle enlargement [16,17]. The progression of these processes can be detected using MRI and considered as a risk factor for the subsequent development of dementia or Alzheimer’s disease [18,19]. Another modifiable risk factor is changes in the parameters of general and biochemical blood tests. It was shown that cardiovascular diseases, untreated diabetes and metabolic syndrome, associated with reduced high-density lipoprotein cholesterol, high glucose and triglycerides levels, may predict the development of AD and dementia [20,21,22]. Moreover, anemia and altered hemoglobin levels were also proved as risk factors [23]. Thus, modifiable risk factors are among the key markers of neurodegeneration and, importantly, can be studied using available laboratory and instrumental methods.

Non-modifiable genetic risk factors contribute significantly to the development of neurodegenerative processes clinically manifested by cognitive decline. Notably, these factors share a similar genetic foundation. For example, the apolipoprotein E gene (*APOE*) has been studied most extensively. The *APOE ε4* allele is associated with the development of both vascular dementia [24] and AD [25]. The presence of the *ε4* allele is considered a major genetic risk factor for AD, increasing the likelihood of the disease threefold in heterozygous and twelvefold in homozygous individuals [26].

However, the *APOE* gene is not the only genetic factor involved in the development of cognitive dysfunctions. Based on summarizing the effects of multiple single nucleotide polymorphisms (SNPs) associated with the development of a particular pathology, assessing polygenic risk scores (PRS) appears to be a promising tool for predicting an individual’s predisposition to diseases and for quantitatively calculating the baseline risk [27,28]. Various studies of PRS models have been associated with the risk of AD and dementia [29] and the progression of MCI into dementia [30]. Additionally, a correlation between PRS of AD and neuroimaging data has been shown [31].

Using additional modifiable factors, such as clinical features, neuroimaging data, biochemical, and immunological parameters of biological fluids, can enhance such predictions in real time. The analysis of dynamic biomarkers can provide further insights into individual risks of dementia development at a certain period of life, enabling timely necessary preventive and therapeutic interventions [32]. In turn, informing the patient about individual risks increases understanding of the necessity for preventive measures and improves patient awareness and adherence to therapy.

Longitudinal studies reflecting the consistent association between changes in biological markers and the progression of cognitive impairments and morphological modifications hold significant diagnostic value.

Previously, our team developed a genetic microarray-based assay of PRS associated with cognitive impairments and AD. For genetic markers, we selected a PRS model consisting of 21 SNPs and the *APOE* gene polymorphism. We demonstrated the differences in PRS values between the control group and dementia patients, using microarray-based assay [33]. The PRS model was validated in the Russian population, showing that individuals in the highest PRS quartile have an increased risk of dementia. The role of the *APOE* gene *ε4* allele was also confirmed. To determine the practical applicability of the developed tool in MCI patients for the individualization of preventive treatment, we conducted a cohort prospective study.

The work aimed to assess the informative role of genetic risk factors on the cognitive dynamics in patients with cognitive impairment and to identify biological factors associated with cognitive disorders in a three-year longitudinal study.

## 2. Materials and Methods

### 2.1. Study Design and Study Population

The study included retired patients over 55 years with MCI who visited the memory clinic, a branch of the Psychiatric Hospital no. 1 Named after N.A. Alekseev of the Department of Health of Moscow, on an outpatient basis with subjective complaints of memory decline, concentration issues, or other manifestations of cognitive deficit. The age range of the participants was between 55 and 93 years, and the mean age was 72 years.

Informed consent was obtained from all participants. The study was conducted under the recommendations of the Helsinki Declaration. Procedures involving human experiments were conducted following the ethical standards of Protocol no. 5 dated 20 September 2020, of the Ethics Committee of the Research Clinical Institute named after L.I. Sverzhevsky of the Moscow Healthcare Department, and Protocol no. 1 dated 25 January 2022, of the Ethics Committee of the State Budgetary Healthcare Institution of Moscow “Psychiatric Hospital no. 1 Named after N.A. Alekseev of the Department of Health of Moscow”.

We assessed three-year dynamics of the cognitive scales of memory clinic patients using the following parameters:

- Socio-demographic factors (age, gender, education, occupational characteristics);
- General and biochemical blood analysis at the first admission;
- Morphological data obtained by MRI at the first admission;
- Genetic markers.

The exclusion criteria included substance abuse and dependence as well as heavy comorbid severe somatic or neurological disorders such as cancer, cirrhosis, chronic lung failure or absence of a lung, tuberculosis, and viral hepatitis. For psychoneurological disorders, exclusion criteria included psychogenic pseudodementia, mental retardation, organic brain diseases, dementia due to schizophrenia, brain injury, epilepsy, tumor, HIV and syphilis, and normotensive hydrocephalus. Additionally, renal or hepatic failure was not permitted.

The diagnosis of MCI was made by an interdisciplinary council consisting of a psychiatrist, a neurologist, and neuropsychologists. The Mini-mental State Examination (MMSE), a neuropsychological scale [34], and the Montreal Cognitive Assessment (MoCA) [35] were used to assess cognitive deficits. Subsequently, a five-week neurorehabilitation course began, during which patients underwent personalized neurocognitive training targeting the most affected cognitive domains with stepwise increasing task complexity, five days a week [36]. Patients received therapeutic physical exercise, information on modifiable risk factors, and individual recommendations for the prevention of cognitive decline. The age range of the participants was between 55 and 93 years, where the mean age was 72 years. We obtained socio-demographic data which included age, gender, education, and occupational characteristics. Occupational conditions were categorized into two types: low-qualified and high-qualified. We defined low-qualified occupation as monotonous work, which does not require intellectual solving of new tasks on a daily basis. Usually, it is not accompanied by the requirement of higher education to apply for such a job.

Participants were subsequently contacted via telephone and invited to a follow-up visit after 36 months, during which the severity of cognitive impairment was assessed. The dynamics of neurocognitive scales were calculated as the difference in total scores between the first visit and the visit after three years. Prior to preventive and rehabilitative therapies, volunteers were recruited and the first visit was evaluated between September 2020 and May 2021. A follow-up appointment was planned ± three months after the 36-month gap. The collection of clinical data was completed in May 2024.

**Figure 1:**
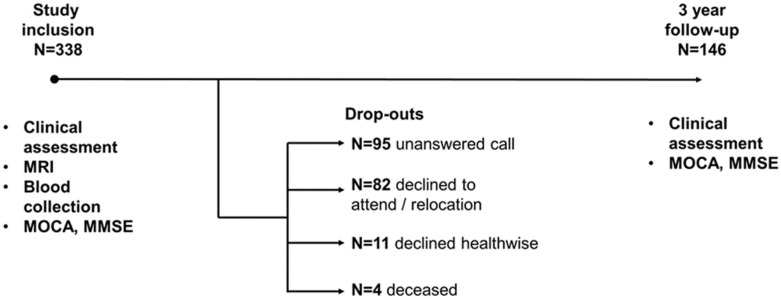
Timeline of research.

The study included 338 patients with MCI, among whom 146 people returned for a follow-up appointment after three years. Among those who dropped out by the third year, 82 refused to return or moved to a different location, 95 repeatedly failed to return phone calls, 11 declined due to health issues, and four patients died (Figure 1).

### 2.2. Blood Collection and Analysis

On the first day of enrollment in the study, blood samples were collected in a fasting state from the cubital vein to obtain plasma and total blood. For further analysis, samples were stored at −80 °C.

Total peripheral blood and biochemical analyses were conducted in the clinical laboratory using hematology analyzer XE-2100 (Sysmex, Kobe, Japan) and ADVIA 120 Hematology System (Siemens, Munich, Germany).

The levels of the following parameters were assessed: cholesterin, triglycerides, HDL, LDL, VLDL, leukocytes, erythrocytes, hemoglobin, hematocrit, platelet count, and glucose.

### 2.3. DNA Extraction and SNP Genotyping of Patients

Genomic DNA was extracted from peripheral blood using a DNA extraction kit with the M-Sorb-Blood reagent for DNA isolation from whole blood on magnetic particles (Syntol Research and Development Company, Moscow, Russia).

The process of identifying 23 SNPs, as well as PRS calculation, is described in detail in our previous study, where the polygenic risk model was tested. The calculation of the PRS mas made using the model proposed by Tosto et al. [37]. The coefficients for the model were obtained from the Polygenic Score (PGS) Catalog (https://www.pgscatalog.org/score/PGS000054/, accessed on 21 August 2024). PRS values were calculated by summing the coefficients multiplied by the number of corresponding effect alleles as follows.

PRS = 0.166 × Nrs6656401_A + 0.199 × Nrs6733839_T + 0.077 × Nrs35349669_T − 0.073 × Nrs190982_G + 0.104 × Nrs9271192_C + 0.095 × Nrs10948363_G − 0.073 × Nrs2718058_G − 0.094 × Nrs1476679_C − 0.105 × Nrs11771145_A + 0.095 × Nrs28834970_C − 0.151 × Nrs9331896_C + 0.077 × Nrs10838725_C − 0.105 × Nrs983392_G − 0.139 × Nrs10792832_A − 0.261 × Nrs11218343_C + 0.131 × Nrs17125944_C − 0.094 × Nrs10498633_T − 0.315 × Nrs8093731_T + 0.14 × Nrs4147929_A − 0.062 × Nrs3865444_A − 0.128 × Nrs7274581_C;

where N represents the number of corresponding effect alleles (from 0 to 2).

For each patient, the *APOE* allele (*ε2/ε3/ε4*) and the PRS value, which was assigned to one of the Q1–Q4 quartiles, were determined [33].

### 2.4. MRI Data

Magnetic resonance imaging data were acquired using an EXCELART Vantage Atlas-X (Toshiba, Minato, Japan) 1.5 T scanner. High-resolution anatomical images were obtained for each study participant based on a T1-weighted sequence: TR = 1200 ms, TE = 50 ms, 200 slices, voxel size: 1 × 1 × 1 mm^3^.

The following data was assessed: total score of GCA, Fazekas, Koedam and MTA scale, third and fourth ventricle width, and hippocampal head, body, and tail height.

### 2.5. Statistical Analysis

Statistical processing was performed using SPSS Statistics version 26.0 (IBM, Endicott, NY, USA) and R software version 4.4.0. The null hypothesis was rejected at a significance level of *p* ≤ 0.05. Mean and standard deviation or median and quartiles were used to describe quantitative variables (if the distribution did not conform to normality), and frequency and proportion (in percent) were used for qualitative variables. The normality of quantitative variable distributions was checked by constructing frequency histograms. For qualitative dependent variables, frequency comparisons between the categories of independent (grouping) variables were performed using Pearson’s χ^2^ test or Fisher’s exact test. For quantitative dependent variables, comparisons were performed using analysis of variance (ANOVA) followed by pairwise comparisons using Dunnett’s method or (if the distribution did not conform to normality) using the Kruskal–Wallis test with pairwise comparisons using the Mann–Whitney test (with Bonferroni correction for multiple comparisons). Pearson’s correlation coefficient was used to identify correlations between quantitative or ordinal variables. A general linear model with repeated measurements was utilized to evaluate the dynamics of MoCA cognitive scores between visits. The MoCA total score served as the dependent variable, while the visit, as well as other variables (such as genetic markers, education level, occupation, etc.), served as the independent variables.

## 3. Results

Three-year dynamics of the cognitive scales were assessed with the following parameters:

- Socio-demographic factors (age, gender, education, and occupational characteristics);
- General and biochemical blood analysis at the first admission;
- Morphological data obtained by MRI at the first admission;
- Genetic markers.

### 3.1. Baseline Characteristics

The design of our study is visually represented in Figure 1. A total of 338 patients participated in the study. Table 1 shows the socio-demographic characteristics of the patients.

Out of the 338 subjects, 146 subjects returned for a follow-up assessment. We detected a statistically significant difference between the initial MoCA neurocognitive scale score and the dropout status in the third year of follow-up (*p* = 0.002). Patients who dropped out had poorer cognitive performance, as measured on the MoCA scale total score. No such correlation was found with the MMSE scale.

### 3.2. Socio-Demographic Factors (Age, Gender, Education, and Occupational Characteristics)

**Figure 2:**
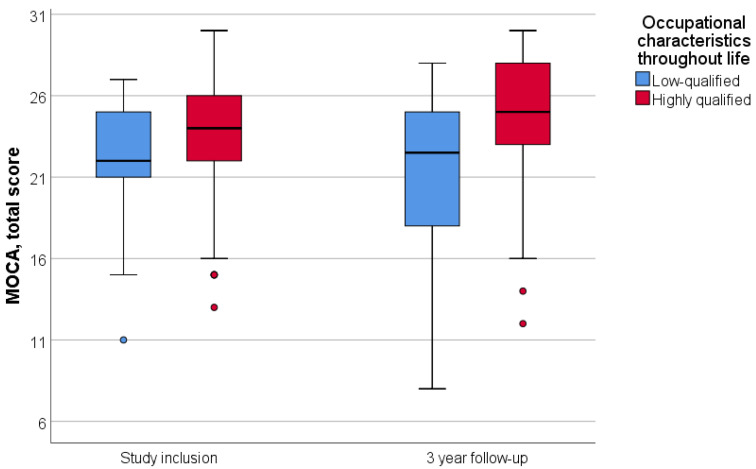
Dependence of dynamics of the MoCA total score on lifetime occupational characteristics. Data are showing median and first and third quartiles using standard boxplot.

Patients were divided into two groups according to their lifetime occupation: highly qualified and low-qualified. Engaging in intellectually demanding labor throughout life was associated with a higher initial MoCA score (*p* = 0.002); however, it did not correlate with the three-year dynamics of neurocognitive scale scores (*p* = 0.575) (Figure 2).

**Figure 3:**
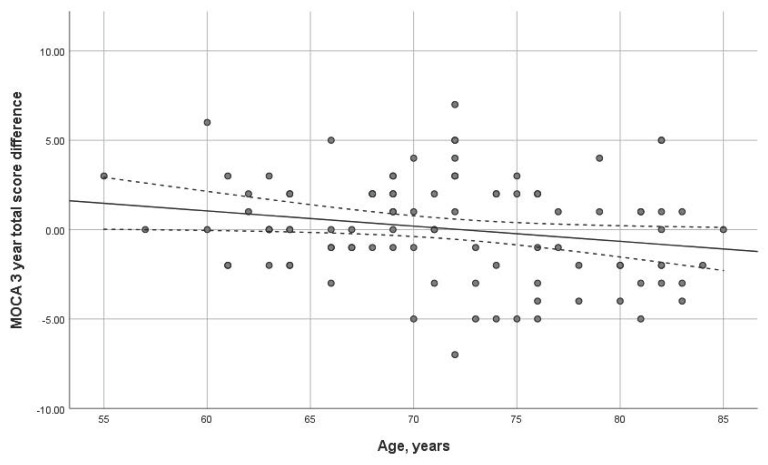
Dependence of dynamics of the total MoCA score on age (*p* = 0.038) over 3-year interval. Trend line with confidence interval is shown, which represents change from first visit to 3-year follow-up.

Age had a statistically significant impact on the three-year dynamics of the MoCA scale (*p* = 0.038). The older the age at the first visit, the more important the MoCA dynamics decline (Figure 3). No significant correlation was found with the MMSE scale.

### 3.3. General and Biochemical Blood Analysis at Initial Admission

Patients underwent general and biochemical blood analyses at the time of inclusion in the study. Table 2 shows the correlation between blood analysis parameters and the dynamics of MoCA and MMSE neurocognitive scales. A correlation was found between cognitive dynamics and red blood cell number and hematocrit level at the first visit, where increased parameters (*p* = 0.011 and *p* = 0.008, respectively) were associated with higher total MoCA scores. The other parameters listed in Table 2 did not correlate with the dynamics of the neurocognitive scales over the three years.

### 3.4. Morphological Data Obtained via MRI at Initial Admission

**Figure 4:**
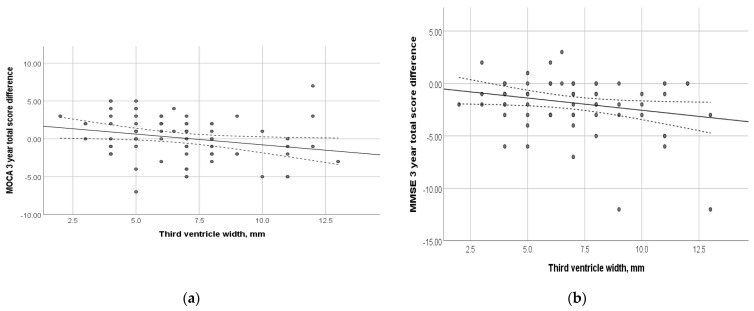
Relationship between the dynamics of cognitive scales and the width of the third ventricle at the time of study inclusion: (**a**) MoCA total score; (*p* = 0.031); (**b**) MMSE total score (*p* = 0.04). Trend line with confidence interval is shown, which represents change from first visit to 3-year follow-up.

At the time of inclusion in the study, the subjects underwent MRI. Figure 4 shows the relationship between the dynamics of the total MoCA and MMSE scores and the width of the third ventricle. Thus, an increase in the width of the third ventricle was shown to be associated with negative cognitive changes (*p* = 0.03 and 0.04, respectively). Among other evaluated morphological parameters, such as the total Fazekas score, MTA, Koedam, GCA, width of the fourth ventricle, and the height of the head, body, and tail of the hippocampus, no correlation with the dynamics of cognitive decline was found (*p* > 0.05) (Supplementary Table S1).

### 3.5. Genetic Markers

We compared MoCA and MMSE scores in patients with MCI at the first visit and after three years and examined the association with genetic variants of *APOE* and PRS scores. Table 3 presents the data on the distribution of quartiles of PRS and *APOE ε4*.

**Figure 5:**
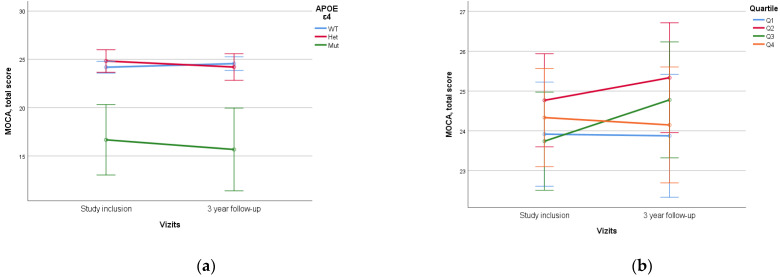
Dependence of 3-year dynamics of MoCA neurocognitive scale scores on *APOE ε4* genetic variant and PRS quartiles ((**a**)—*APOE ε4*, *p* < 0.005, (**b**)—PRS quartiles, *p* = 0.197). The PRS value was calculated using a model containing 23 SNPs. WT—wild-type, het—*ε4*-heterozygous, mut—mutant, *ε4*—homozygous. Data are presented as M ± SD.

When assessing *APOE* alleles, a statistically significant difference (*p* < 0.005) was found in the dynamics of MoCA and MMSE scores between patients with *ε4/ε4* homozygote and those with other variants, which was characterized by lower initial neurocognitive scales scores and higher rates of decline. No statistical difference was found between *ε4* heterozygotes and homozygotes without *ε4* (Figure 5).

**Figure 6:**
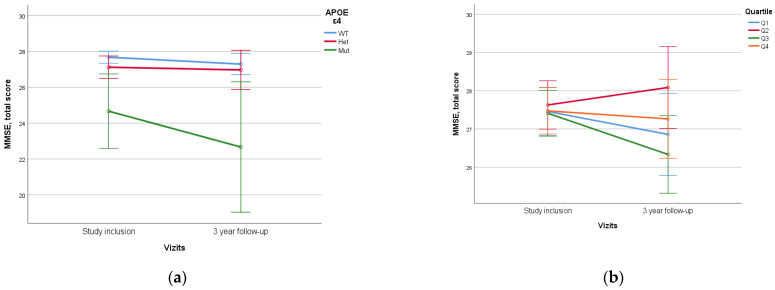
Dependence of 3-year dynamics of MMSE neurocognitive scale scores on *APOE ε4* genetic variant and PRS quartiles ((**a**)—*APOE ε4*, *p* = 0.012, (**b**)—PRS quartiles, *p* = 0.310). The PRS value was calculated using a model containing 23 SNPs. WT—wild-type, het—*ε4*-heterozygous, mut—mutant, *ε4*—homozygous. Data are presented as M ± SD.

No statistically significant difference was identified when assessing PRS value and quartile stratification of PRS (Figure 6).

In our study, we evaluated the dependence of dynamics of cognitive dysfunction on general and biochemical blood analysis indicators, and morphological and genetic parameters and their assessed potential as predictors of cognitive decline. As biomarkers of progression of cognitive impairment in blood analysis, we assessed the total blood count and biochemical parameters related to lipid and carbohydrate metabolism. A correlation was found between decreased erythrocyte number and hematocrit levels with declining cognitive performance. When analyzing the MRI data, the width of the third ventricle was associated with MoCA cognitive scale dynamics. Genetic markers included *APOE* gene alleles and PRS quartiles. We found that the *APOE ε4/ε4* variant was associated with lower initial cognitive scale scores and with negative cognitive performance dynamics.

## 4. Discussion

In this study, we tracked the three-year dynamics of cognitive impairments in MCI patients who sought outpatient care for cognitive decline. During this period, 57% of subjects dropped out of the study. According to our results, patients with initially lower MoCA total scores were less likely to return for follow-up assessments after three years. Thus, patients with more severe cognitive decline were more likely to drop out, which could have inflated MoCA scores and represents a substantial limitation of our study.

We evaluated the impact of socio-demographic factors on cognitive performance dynamics. It is known that cognitive decline is directly associated with age [38]. Thus, in our work, older age was associated with negative dynamics in the MoCA total score.

We also compared the participants’ lifetime job characteristics and level of education. We discovered a correlation between lifetime intellectual work and higher MoCA scores when compared to low-skilled jobs. These findings support theories of different cognitive reserves based on lifestyle [39]. However, no significant difference in the rate of cognitive decline progression was identified between these two groups.

In addition to lifestyle factors, other factors significantly influence cognitive decline progression. Previously, we compared the concentration of inflammatory markers and lipid metabolism parameters in patients with dementia and MCI [40]. In this study, we assessed the impact of blood parameters routinely performed in clinics on cognitive decline progression. We found, consistent with the data linking neurodegenerative processes with anemia, an association between cognitive decline and blood laboratory parameters. As per Faux et al. [41], it was shown that individuals with AD display reduced levels of laboratory markers that signify anemia. These markers include decreased mean hemoglobin concentration, decreased erythrocyte volume, and increased erythrocyte sedimentation rate. Low hematocrit, erythrocyte count, and hemoglobin were also discovered to be linked to a higher risk of MCI [42,43]. Our data indicate that low hematocrit and total RBC count positively correlate with the dynamics of cognitive decline. We believe that these parameters, which are routinely assessed in clinical practice, could be included in risk prediction panels for worsening cognitive dysfunction symptoms due to their ubiquitous applicability.

Another blood-related risk factor for dementia, according to some researchers, is the assessment of lipid and carbohydrate metabolism [44]. However, our study did not find any associations between various lipid metabolism indicators, glucose levels, and the dynamics of cognitive impairments. This may be due to the specific characteristics of our sample. Some somatic diseases, which we did not account for in our work, can influence these parameters. Perhaps, a larger homogeneous sample would reveal an association between lipid and carbohydrate parameters and cognitive dynamics.

Numerous studies have focused on brain morphology in cognitive decline. Thus, it has been shown that even before the dementia stage, signs of brain atrophy are visible [45]. Our MRI data confirm the relationship between atrophy and neurodegenerative processes and, consequently, cognitive decline. Reus et al. suggest using a multimodal risk assessment of MCI progression in AD, which includes MRI neuroimaging data alongside genetic features [46]. Based on the results of our study, assessing the width of the third ventricular is one of the most important and promising characteristics of MRI data. Previous data highlighted the importance of using ventricular volume as a prognostic marker of MCI progression to dementia [47,48]. In our study, the correlation found using a 1.5T MRI scanner was weak. This might be related to the scanner’s resolution. We believe that further research investigating this correlation using a higher-resolution scanner is necessary. For example, Chow et al. compared the effectiveness of 1.5T and 3T MRI scanners in detecting the degree of brain atrophy, showing the feasibility and greater effectiveness of using 3T MRI [49].

Previously, we conducted a study on the impact of biological factors on the course of MCI and the effectiveness of neurorehabilitation, which confirmed the significant contribution of the genetic profile in assessing the success of preventive therapy [50]. One effective method for investigating the influence of genetic factors on the development and course of polyethiological diseases is the construction of models of PRS, including several genetic variants (SNPs) associated with the pathology. Our research group recreated a PRS model for dementia development based on the Tosto PRS model, which includes 21 SNPs [37]. Previously, we published a study showing its effectiveness in comparing patients with dementia and healthy controls [33]. In this work, we further evaluated the contribution of PRS to the development of cognitive deficits in a longitudinal study and expanded our diagnostic search to include modifiable risk factors alongside *APOE* assessment. One of the main genetic predictors of cognitive impairment is the *APOE* gene *ε4* allele, as demonstrated in large GWAS studies [51]. Therefore, in our work, we also evaluated the impact of the *APOE* genetic variant on the dynamics of overall MoCA and MMSE scores. Our results show that the *APOE ε4/ε4* genotype was associated with statistically significantly lower initial total neurocognitive scores and a higher rate of their decline over time. Notably, these results were obtained although this genetic variant was present in only 3 out of 146 individuals in our study. This aligns with global statistics, where homozygous *APOE ε4/ε4* carriers are found in the population in less than 2% of cases [52]. These results may be explained by the identification of dementia cases associated with homozygous *APOE ε4*-carrying as a distinct genetically determined disease [53].

It should be noted that the scientific literature contains data indicating an increased rate of cognitive decline and a higher risk of transition to dementia in individuals with the heterozygous variant of the *APOE* allele [54], which was not observed in the present study.

No significant association of the PRS with cognitive dynamics over three years was found in our study. The differences in MoCA and MMSE scores were statistically insignificant, considering the values of Q4 PRS and both homozygous and heterozygous *APOE* states. At the same time, Jung-Min Pyun et al. found an association between MCI progression to dementia when assessing the PRS, but their PRS model assessed 426 genes and the duration of follow-up was five years [30]. This may indicate that it is necessary to refine the model and include additional genes or factors, but the influence of neurocognitive rehabilitation may also be an important contributing factor. The lack of statistically significant results between non-carriers and *APOE* single *ε4*-carriers, a known risk factor for cognitive decline [54], may also indicate that the rehabilitation program is effective and may delay cognitive decline or prevent it to some degree. Certainly, studies with a longer observation period are necessary to draw a reliable conclusion about the influence of genetic factors on the dynamics of cognitive functions in patients with MCI. On the other hand, improvements in bioinformatics approaches offer hope for the development of polygenic models that will better predict the risks and prognosis for individuals associated with neurodegeneration.

The studied patient group was observed at the Memory Clinic, informed about individual risk factors, and educated on cognitive decline prevention. They also received current non-drug treatments, including neurocognitive training and therapeutic physical exercises. This could have delayed the onset of cognitive dysfunction symptoms, and the selected three-year interval might be insufficient to detect a statistically significant difference in cognitive decline for both PRS and *APOE* genotypes other than homozygous *ε4*/*ε4*. The effectiveness of the described neurocognitive training has already been demonstrated in our previous study. Thus, neurocognitive rehabilitation can potentially delay the progression of serious cognitive impairments, despite of the genetic risks [50]. Several researchers also emphasize that lifestyle impacts the risk of dementia, regardless of the *APOE* variant [55] and PRS [54]. Additionally, we note that due to the more frequent dropout of subjects with low initial MoCA scores, our study might have less frequently accounted for individuals with the most negative dynamics.

It is crucial to emphasize the importance of conducting longitudinal studies when assessing the effectiveness of various markers in predicting the development of dementia and evaluating the rate of cognitive decline. Previously, other authors have shown the assessment of the degree of MCI progression to dementia based on the PRS indicators [30]. We believe that more research is needed to assess not only the progression to dementia but also a detailed analysis of the dynamics of cognitive metrics, as a decline is peculiar to every elderly person.

## 5. Conclusions

Our study showed a significant association between the rate of cognitive decline and carrying a homozygous *APOE ε4* variant, as well as MRI indicators of the third ventricular width and parameters of body oxygenation assessment. However, the selected PRS model did not predict the progression of cognitive decline at the studied time points. This may be due to the insufficient duration of the study period and the characteristics of our sample (i.e., sample size, dropout of patients with initially low MoCA scores, prevention and therapy). Furthermore, not just genetic variables, such as *APOE* and PRS, may be predictive of the progression of cognitive decline. Blood parameters related to body oxygenation and neuroimaging data can help analyze the overall picture. This work indicates that a three-year interval might be insufficient for assessing cognitive dynamics among individuals who regularly manage their health and adhere to medical advice. Identifying a representative sample remains a challenge for prospective cognitive studies, requiring subjects’ compliance and involvement.

## Figures and Tables

**Table 1:** Socio-demographic characteristics of the patients; *p*-value between dropouts and non-dropouts.

	All Subjects (*N* = 338)	Dropouts (*N* = 192)	Non-Dropouts (*N* = 146)	*p*-Value
Age (Mean ± SD)	71.2 ± 7.5	71.4 ± 7.9	70.9 ± 7.1	*p* = 0.511
Years of secondary education (Mean ± SD)	11.2 ± 1.6	11.2 ± 1.7	11.2 ± 1.4	*p* = 0.85
Years of higher education (if any) (Mean ± SD)	4.6 ± 2.0	4.4 ± 2.5	5.0 ± 1.1	*p* = 0.01 **
Initial MoCA score (Median; Mean ± SD)	24; 23.4 ± 3.6	23; 23.1 ± 3.5	25; 23.8 ± 3.6	*p* = 0.013 **
Initial MMSE score (Median; Mean ± SD)	27; 27.2 ± 2.1	27; 27.0 ± 2.2	27; 27.5 ± 1.9	*p* = 0.033 **
Gender	Female 84.0% (*N* = 284) Male 16.0% (*N* = 54)	Female 82.3% (*N* = 158) Male 17.7% (*N* = 34)	Female 86.3% (*N* = 126) Male 13.7% (*N* = 20)	*p* = 0.319
Higher education attainment	Yes 57.4% (*N* = 187) No 42.6% (*N* = 139)	Yes 53.3% (*N* = 97) No 46.7% (*N* = 85)	Yes 62.5% (*N* = 90) No 37.5% (*N* = 54)	*p* = 0.149
Occupational characteristics throughout life	Highly qualified 81.8% (*N* = 265) Low-qualified 18.2% (*N* = 59)	Highly qualified 75.6% (*N* = 136) Low-qualified 24.4% (*N* = 44)	Highly qualified 89.6% (*N* = 129) Low-qualified 10.4% (*N* = 15)	*p* = 0.002 **

**—*p* value ≤ 0.05.

**Table 2:** Association of neurocognitive scale dynamics with blood analysis indicators.

Characteristics	Dynamics of the MoCA Scale		Dynamics of the MMSE Scale	
	Pearson Correlation	*p* Value	Pearson Correlation	*p* Value
Cholesterin	−0.016	0.877	−0.099	0.346
Triglycerides	0.063	0.544	0.016	0.878
HDL	−0.122	0.24	−0.11	0.295
LDL	−0.038	0.719	−0.073	0.494
VLDL	0.065	0.536	0.018	0.868
Leukocytes	0.148	0.356	−0.197	0.229
Erythrocytes	0.396 **	0.011 **	−0.194	0.243
Hemoglobin	0.118	0.47	−0.064	0.703
Hematocrit	0.412 **	0.008 **	−0.096	0.564
Platelet count	0.051	0.757	0.022	0.895
Glucose	−0.127	0.222	−0.172	0.101

**—*p* value ≤ 0.05.

**Table 3:** Genetic characteristics of the sample.

	All Subjects (*N* = 338)	Dropouts (*N* = 192)	Non-Dropouts (*N* = 146)
PRS	Q1 24.9% (*N* = 84)	Q1 25.5% (*N* = 49)	Q1 24.0% (*N* = 35)
	Q2 25.2% (*N* = 85)	Q2 26.0% (*N* = 50)	Q2 24.0% (*N* = 35)
	Q3 25.7% (*N* = 87)	Q3 25.5% (*N* = 49)	Q3 26.0% (*N* = 38)
	Q4 24.3% (*N* = 82)	Q4 22.9% (*N* = 44)	Q4 26.0% (*N* = 38)
*APOE ε4*	wild-type 76.3% (*N* = 258)	wild-type 77.1% (*N* = 148)	wild-type 75.3% (*N* = 110)
	*ε4*-heterozygous 21.9% (*N* = 74)	*ε4*-heterozygous 21.4% (*N* = 41)	*ε4*-heterozygous 22.6% (*N* = 33)
	mutant *ε4*-homozygous 1.8% (*N* = 6)	mutant *ε4*-homozygous 1.8% (*N* = 3)	mutant *ε4*-homozygous 2.0% (*N* = 3)

## Data Availability

Data available on request due to restrictions privacy.

## Supplementary Materials

The following supporting information can be downloaded at: https://www.mdpi.com/article/10.3390/diagnostics14171883/s1, Figure S1: Value of the total MoCA score at the first visit in dropout and non-dropout patients; Table S1: Correlation of MoCA and MMSE scale dynamics with MRI scores at the first visit.
