# Supplementary material for: Dynamics of Cognitive Impairment in MCI Patients over a Three-Year Period: The Informative Role of Blood Biomarkers, Neuroimaging, and Genetic Factors
Source: Diagnostics (Basel). 2024 Aug 28;14(17):1883. doi: 10.3390/diagnostics14171883 (PMC11394601; doi:10.3390/diagnostics14171883)
Supplement: Supplementary file 1 [file diagnostics-14-01883-s001.zip › diagnostics-3151841-supplementary.pdf]

Supplementary Materials:

Table S1. Correlation of MoCA and MMSE scale dynamics with MRI scores at the first visit.

	MoCA scale (P value)	MoCA scale (Pearson correlation)	MMSE scale (P value)	MMSE scale (Pearson correlation)
Total score GCA scale	0,267	-0,129	0,062	-0,218
Total score Fazekas scale	0,752	-0,037	0,363	-0,107
Total score Koedam scale	0,705	0,044	0,978	0,003
Total score MTA scale	0,459	-0,086	0,306	-0,121
Third ventricle width	**0,031**	**-0,248****	**0,040**	**-0,240****
Fourth ventricle width	0,115	-0,182	0,354	-0,109
Hippocampal head height on the right	0,930	0,010	0,181	0,157
Hippocampal head height on the left	0,848	-0,022	0,105	0,190
Hippocampal body height on the right	0,591	0,063	0,074	0,209
Hippocampal body height on the left	0,339	0,111	0,061	0,219
Hippocampal tail height on the right	0,225	0,141	0,403	0,099
Hippocampal tail height on the left	0,138	0,172	0,301	0,122

** - P value ≤ 0.05

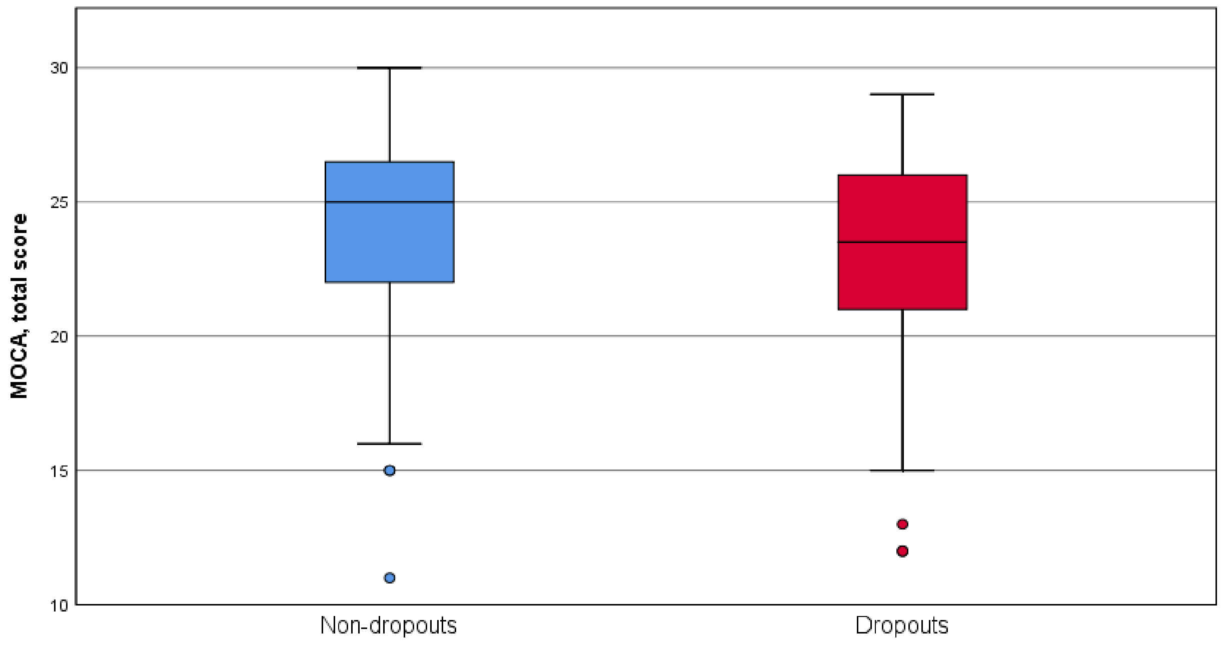

Figure S1. Value of the total MoCA score at the first visit in dropout and non-dropout patients
